# Adverse outcome pathway for pregnane X receptor-induced hypercholesterolemia

**DOI:** 10.1007/s00204-023-03575-4

**Published:** 2023-08-29

**Authors:** Anna Itkonen, Jukka Hakkola, Jaana Rysä

**Affiliations:** 1 School of Pharmacy, Faculty of Health Sciences, University of Eastern Finland, P.O. Box 1627, 70211 Kuopio, Finland; 2 Research Unit of Biomedicine and Internal Medicine, Biocenter Oulu, Medical Research Center Oulu, University of Oulu and Oulu University Hospital, Oulu, Finland

**Keywords:** AOP, Cholesterol, Hypercholesterolemia, PXR, SREBP2, PCSK9

## Abstract

Pharmaceuticals and environmental contaminants contribute to hypercholesterolemia. Several chemicals known to cause hypercholesterolemia, activate pregnane X receptor (PXR). PXR is a nuclear receptor, classically identified as a sensor of chemical environment and regulator of detoxification processes. Later, PXR activation has been shown to disrupt metabolic functions such as lipid metabolism and recent findings have shown PXR activation to promote hypercholesterolemia through multiple mechanisms. Hypercholesterolemia is a major causative risk factor for atherosclerosis and greatly promotes global health burden. Metabolic disruption by PXR activating chemicals leading to hypercholesterolemia represents a novel toxicity pathway of concern and requires further attention. Therefore, we constructed an adverse outcome pathway (AOP) by collecting the available knowledge considering the molecular mechanisms for PXR-mediated hypercholesterolemia. AOPs are tools of modern toxicology for systematizing mechanistic knowledge to assist health risk assessment of chemicals. AOPs are formalized and structured linear concepts describing a link between molecular initiating event (MIE) and adverse outcome (AO). MIE and AO are connected via key events (KE) through key event relationships (KER). We present a plausible route of how PXR activation (MIE) leads to hypercholesterolemia (AO) through direct regulation of cholesterol synthesis and via activation of sterol regulatory element binding protein 2-pathway.

## Background

Hypercholesterolemia is a lipid metabolism disorder, defined by high level of low-density lipoprotein (LDL) cholesterol in the blood. Hypercholesterolemia is a major contributor to the development of atherosclerosis, the leading cause of mortality in developed countries (Ziegler et al. 2020). The risk factors for hypercholesterolemia include sedentary lifestyle, western diet, obesity as well as genetic predisposition (Garg and Simha 2007; Ziegler et al. 2020). In addition, pharmaceuticals and environmental chemicals elevate lipid levels and in fact more than hundred drugs increasing total cholesterol levels have been identified (Karpale et al. 2022). However, the mechanisms underlying chemical-induced hypercholesterolemia remain poorly understood. Interestingly, several drugs, which increase lipid levels, are known to activate pregnane X receptor (PXR, NR1I2) (Karpale et al. 2022).

PXR is a sensor of xenobiotics, detecting fluctuations in chemical environment (Blumberg et al. 1998; Kliewer et al. 1998). PXR recognizes various structurally diverse endogenous and exogenous substances as ligands in a species-specific manner (Kliewer et al. 1998). Known ligands for PXR include pharmaceuticals (e.g., rifampicin, phenobarbital, clotrimazole) (Lehmann et al. 1998), pesticides (e.g., metolachlor, propiconazole, permethrin pyrethroid) and environmental contaminants such as polychlorinated biphenyls (Lemaire et al. 2006). In addition to the originally identified function as a regulator of detoxification functions, PXR activation has been found to disturb cardiometabolic functions such as glucose tolerance, lipid metabolism and regulation of blood pressure (Hukkanen and Hakkola 2020).

Cholesterol homeostasis in mammalian cells is maintained by a feedback system depending on cholesterol level and modulating the transcription of genes responsible for cholesterol synthesis and uptake (Brown and Goldstein 1997). A family of transcription factors called sterol regulatory element binding proteins (SREBPs) are the master regulators of the feedback system. SREBP pathway activity is regulated by cellular sterol level. In the condition of high sterol level, SREBPs remain inactive, the expression of the target genes is low, and consequently cholesterol synthesis is repressed. Recently, PXR was shown to increase lipid levels by SREBP-mediated mechanism inducing genes participating in cholesterol synthesis and uptake (Karpale et al. 2021). Furthermore, PXR has been shown to directly regulate at least one of the cholesterol synthesis genes (Gwag et al. 2019).

We applied an adverse outcome pathway (AOP) framework to elucidate the mechanisms linking PXR activation to increased level of plasma LDL cholesterol. AOPs are formalized and structured linear concepts connecting a molecular initiating event (MIE) to an adverse outcome (AO) via key events (KE) and key event relationships (KER) (Ankley et al. 2010). AOP summarizes existing knowledge to support health risk assessment of chemicals. AOPs do not describe complex cellular or molecular mechanisms but rather are simplified versions of toxicity pathways focusing on essential events (Svingen et al. 2021). Based on AOP-wiki database (https://aopwiki.org/events/245 accessed 25 Nov 2022), PXR activation has been proposed as an MIE in two AOPs, but neither of them links PXR activation to hypercholesterolemia. Here, we describe strategy of AOP development, which aims to explain the observed AO in terms of a MIE and KEs (Villeneuve et al. 2014). The AOP was developed according to the “Users' Handbook supplement to the Guidance Document for developing and assessing Adverse Outcome Pathways”, issued by the Organisation for Economic Co-operation and Development the users’ handbook (OECD 2018).

## Proposed mechanism for PXR-induced hypercholesterolemia

**Fig. 1:**
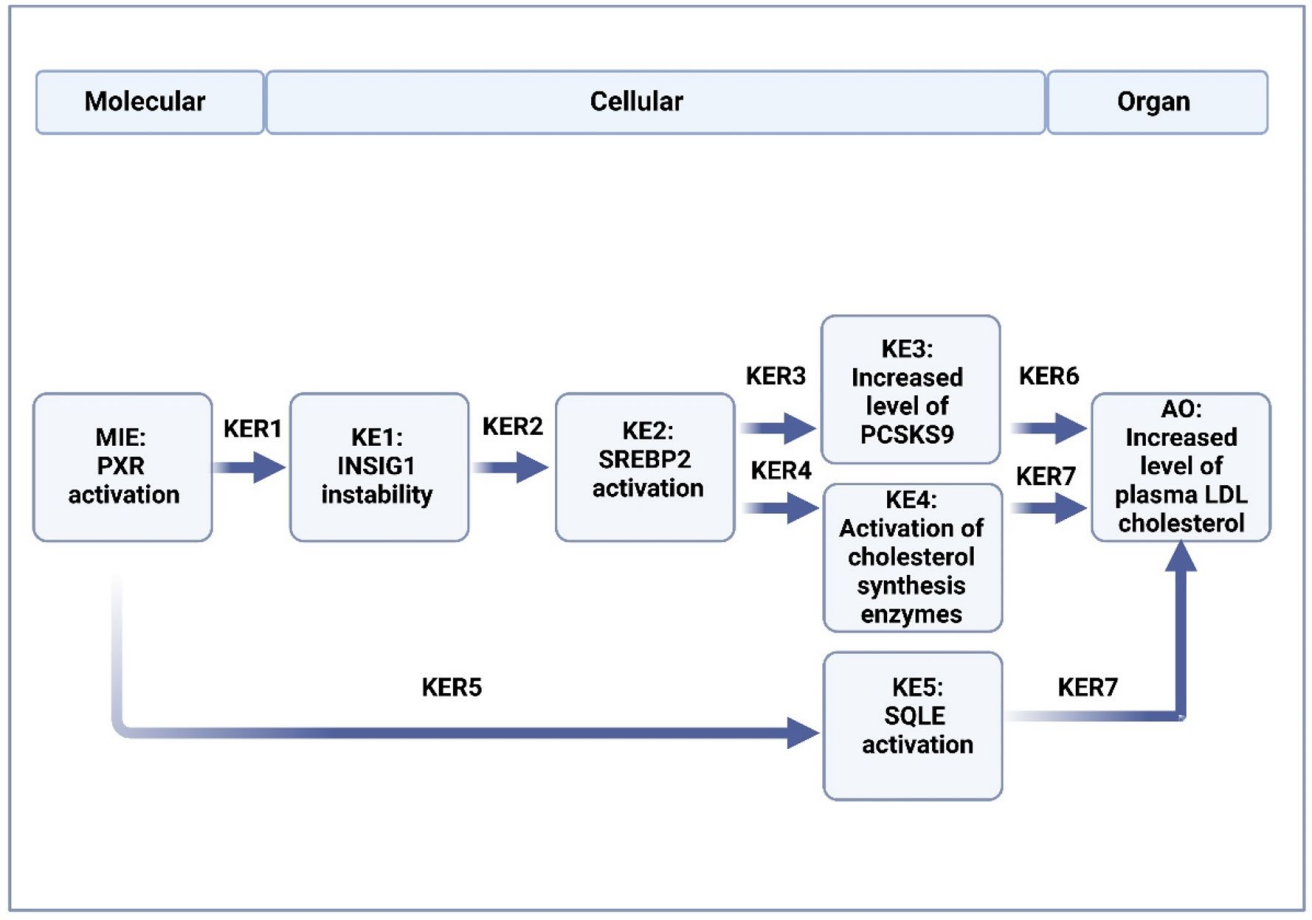
Graphic representation of the adverse outcome pathway from PXR activation to increased level of plasma LDL cholesterol. The molecular initiating event (MIE) is activation of pregnane X receptor (PXR), which leads to the first key event (KE), unstabilized structure of insulin-induced gene (INSIG1). Unstable structure of INSIG1 allows activation of sterol regulatory element binding protein 2 (SREBP2), the second KE in the pathway. Activation of SREBP2 increases the transcription of its target genes, leading to increased synthesis and activation of proprotein convertase subtilisin kexin type 9 (PCSK9) (KE3) and enzymes participating in cholesterol synthesis (KE4). PXR directly activates squalene epoxidase (SQLE) (KE5), rate-limiting enzyme of cholesterol synthesis. Increased serum level of PCSK9 leads to decreased amount of low-density lipoprotein (LDL) receptors, causing diminished uptake of LDL from circulation by liver. Simultaneously, activation of cholesterol synthesis enzymes enhances cholesterol synthesis. These mechanisms lead to increased level of plasma LDL cholesterol, the adverse outcome (AO) of the pathway. Arrows represent direct key event relationships (KERs) that link the KEs

Binding of a suitable ligand activates PXR, being the MIE of the AOP (Fig. 1.). PXR activation leads to destabilized structure of insulin-induced gene 1 (INSIG1) protein, the first KE. Unstable structure of INSIG1 allows cholesterol-independent activation of sterol regulatory element binding protein 2 (SREBP2) which is the second KE in the pathway. This further increases the transcription of SREBP2 target genes, leading to increased synthesis and activation of proprotein convertase subtilisin kexin type 9 (PCSK9) (KE3) and enzymes participating in cholesterol synthesis (KE4). Moreover, PXR is able to directly activate squalene epoxidase (SQLE) (KE5), the rate-limiting enzyme of cholesterol synthesis. Increased level of PCSK9 in serum leads to decreased amount of LDL receptors (LDLR) in hepatocyte plasma membrane, causing diminished uptake of LDL from circulation by liver. Simultaneously, activation of cholesterol synthesis enzymes enhances cholesterol synthesis. Together these mechanisms lead to increased level of plasma LDL cholesterol, which is the adverse outcome (AO) of the pathway.

## MIE: PXR activation (KE:245)

### Key event description

The MIE, activation of PXR (NR1I2), is already described in AOP-Wiki as a KE (KE:245, Activation PXR/SXR https://aopwiki.org/events/245 accessed 25 Nov 2022). PXR recognizes various structurally divergent endogenous and exogenous ligands (di Masi et al. 2009) due to its large, flexible, and hydrophobic ligand binding domain (Zhou et al. 2009b). Upon ligand binding, PXR forms a heterodimer with another nuclear receptor, retinoid X receptor. The heterodimer then binds to specific promoter sequences regulating transcription of various genes involved in cellular metabolism and clearance of xenobiotics and endotoxins in the liver and intestine such as genes encoding cytochrome P450 enzymes and transporter proteins (Kliewer et al. 1998; Zhou et al. 2009b; Hakkola et al. 2016). PXR also interacts with other transcription factors, which increases the complexity of regulatory networks (Hakkola et al. 2016).

### Domain of applicability

PXR expression is well established in humans and rodents, and *PXR* gene has been characterized in several vertebrate species including zebrafish, frog, chicken, dog, pig, and rhesus monkey (Jones et al. 2000; Moore et al. 2002). PXR is expressed mainly in liver and intestine, and in low levels in lungs, stomach, peripheral blood monocytes, the blood–brain barrier, uterus, ovary, placenta, breast, osteoclasts, heart, adrenal gland, bone marrow, and certain regions of the brain (Kliewer et al. 1998; Lamba et al. 2004; Daujat-Chavanieu and Gerbal-Chaloin 2020). Expression of PXR increases through life stages starting with low expression level during fetal and neonatal stages and reaching the highest expression level in the adulthood (Daujat-Chavanieu and Gerbal-Chaloin 2020). Despite the low expression level during fetal development, the activity of PXR is induced by PXR ligands in fetal liver (Xiang et al. 2020; Dai et al. 2021).

The DNA-binding domain of PXR is highly conserved, but the ligand-binding domain differs between species (Jones et al. 2000). Consequently, the ligand preference of PXR differs significantly between species (Kliewer et al. 1998). Additionally, circadian variation and sex dimorphism of PXR have been observed in murine models (Wolbold et al. 2003; Lu et al. 2013; Xiang et al. 2020; Dai et al. 2021). Sex-specific activation of PXR has also been detected in human hepatic cell lines, whereas the findings of sex-dependent expression of *PXR* mRNA in human liver samples are inconsistent (Wolbold et al. 2003; Lamba et al. 2004; Xiang et al. 2020).

## KER1 between PXR activation and stability of INSIG1

### Key event relationship description

Recently, PXR activation was suggested to destabilize the structure of INSIG1 protein (Karpale et al. 2021). Previously, PXR activation has been shown to alter the expression of *Insig1* in vivo and in vitro (Roth et al. 2008; Zhou et al. 2009a; Farmahin et al. 2019; Knebel et al. 2019). Insulin-induced genes (*INSIG1* and *INSIG2*) code endoplasmic reticulum (ER) membrane proteins with similar functions (Yang et al. 2002; Yabe et al. 2002). INSIG1 maintains the intracellular lipid metabolism homeostasis in hepatocytes and adipocytes by regulating de novo synthesis and uptake of cholesterol and fatty acids (Ouyang et al. 2020). Therefore, deviation in expression or function of INSIG1 is linked with the pathogenesis of lipid disorders. Due to its crucial role in lipid metabolism, INSIG1 is highly expressed in hepatocytes and adipocytes (Diamond et al. 1993; Peng et al. 1997). In humans and rodents, INSIG1 is mostly expressed in the liver, but at low levels also in other tissues (Peng et al. 1997; Uhlén et al. 2015). The conservation of INSIG1 sequence varies among vertebrate species (mouse, hamster, zebrafish) between 70 and 92% compared to human (Yabe et al. 2002). The variation is mostly exerted in the hydrophilic NH_2_- and COOH-terminal sequences. INSIG1 is expressed already during development (Iritani et al. 1993; Lou et al. 2014).

### Biological plausibility

Biological plausibility for PXR activation leading to altered function of INSIG1 is moderate. PXR is shown to bind to DR-4 site in the upstream promoter region of INSIG1 and induce *INSIG1* mRNA expression (Roth et al. 2008). The mechanism causing destabilization of INSIG1 protein is currently unknown.

### Empirical evidence

The ability of PXR to increase (Roth et al. 2008; Zhou et al. 2009a; Knebel et al. 2019) or decrease (Farmahin et al. 2019) *Insig1* mRNA expression is described in few studies characterizing the effects of PXR on INSIG1 in rodents and hepatic cells in vitro. Recently, Karpale et al. (2021) reported increased *Insig1* mRNA expression after PXR activation in livers of obese male C57BL/6 N mice treated with a rodent PXR ligand, pregnenolone 16α-carbonitrile (PCN); this induction was not seen in *Pxr* knockout mice (Karpale et al. 2021). On the contrary, PXR did not affect the protein level of INSIG1, despite cholesterol accumulation, which should enhance INSIG1 production and stabilize the protein structure. The authors concluded that PXR may alter the proteolytic machinery controlling INSIG1 stability (Karpale et al. 2021). Alternatively, the INSIG1 translation could be repressed.

### Uncertainties and inconsistencies

Even though activation of PXR is observed to alter *Insig1* expression on mRNA level, there is only one study determining the effects on protein level or further on INSIG1 stability (Table 1). In addition, the mechanism by which PXR would alter the stability of INSIG1 is unknown.

**Table 1:** Quality of evidence rating for KERs

Observations	Model organism			Level of observation		Type of study		Citation
	Hu	Mo	Ha	mRNA	Protein	1	2	
PXR activation leads to instability of INSIG1 (KER1)		X		X	X		X	(Karpale et al. 2021)
Degradation of INSIG1 leads to SREBP2 activation (KER2)	X	X	X	X	X	X	X	(Yang et al. 2002; Yabe et al. 2002; Engelking et al. 2005; McFarlane et al. 2014)
SREBP2 activation leads to increased level of PCSK9 (KER3)	X	X		X	X	X	X	(Horton et al. 2003; Maxwell et al. 2003; Hyun et al. 2008; Karpale et al. 2021)
SREBP2 activation leads to increased level of cholesterol synthesis enzymes (KER4)	X	X		X	X	X	X	(Horton et al. 2003; Maxwell et al. 2003; Howe et al. 2017; Karpale et al. 2021)
PXR activation leads to increased activity of SQLE (KER5)	X	X		X	X	X	X	(Gwag et al. 2019; Jiang et al. 2021; Karpale et al. 2021)
Increased level of PCSK9 leads to increased level of plasma LDL cholesterol (KER6)	X	X	X	X	X		X	(Benjannet et al. 2004; Lalanne et al. 2005; Lambert et al. 2006, 2008; Grefhorst et al. 2008; Dong et al. 2010)
Increased level of cholesterol synthesis enzymes leads to increased level of plasma LDL cholesterol (KER7)	X	X		X	X	X	X	(Engelking et al. 2005; McFarlane et al. 2014; Gwag et al. 2019)

*Hu* human; *Mo* mouse; *Ha* Hamster; *1* in vitro; *2* in vivo; *PXR* pregnane X receptor; *INSIG1* insulin-induced gene 1; *SREBP2* sterol regulatory element binding protein 2; *PCSK9* proprotein convertase subtilisin kexin type 9; *SQLE* squalene epoxidase; *LDL* low-density lipoprotein

## KER2 between INSIG1 stability and SREBP2 activation

### Key event relationship description

INSIG1 controls cholesterol synthesis and uptake via regulating the activation of SREBP2 in a cholesterol-dependent fashion by binding to SREBP cleavage-activating protein (SCAP) (Brown and Goldstein 1999). Binding of INSIG1 to SCAP promotes the retention of SREBP2 in ER and inhibits transfer to Golgi and activation of SREBP2.

The SREBP family of membrane-bound transcription factors consists of three members, SREBP1a, SREBP1c, and SREBP2 (Brown and Goldstein 1997). Cholesterol metabolism is regulated especially by SREBP2. The three members of SREBPs are expressed ubiquitously in several mammalian species and are detected in all tissues (Horton et al. 2002; Eberlé et al. 2004). SREBP1a and SREBP2 are the predominant isoforms in majority of cell lines, whereas SREBP1c and SREBP2 are the most abundant isoforms in the liver of mice and human (Shimomura et al. 1997). SREBP1a and -1c are produced by *SREBPF1* and SREBP2 by *SREBPF2* genes, respectively (Hua et al. 1995; Miserez et al. 1997) and share 47% homology with each other (Eberlé et al. 2004). SREBP2 seems to have a crucial role already in the early stages of development, since deletion of *Srebpf2* gene is observed to be lethal in animal models (Shimano et al. 1997).

### Biological plausibility

**Fig. 2:**
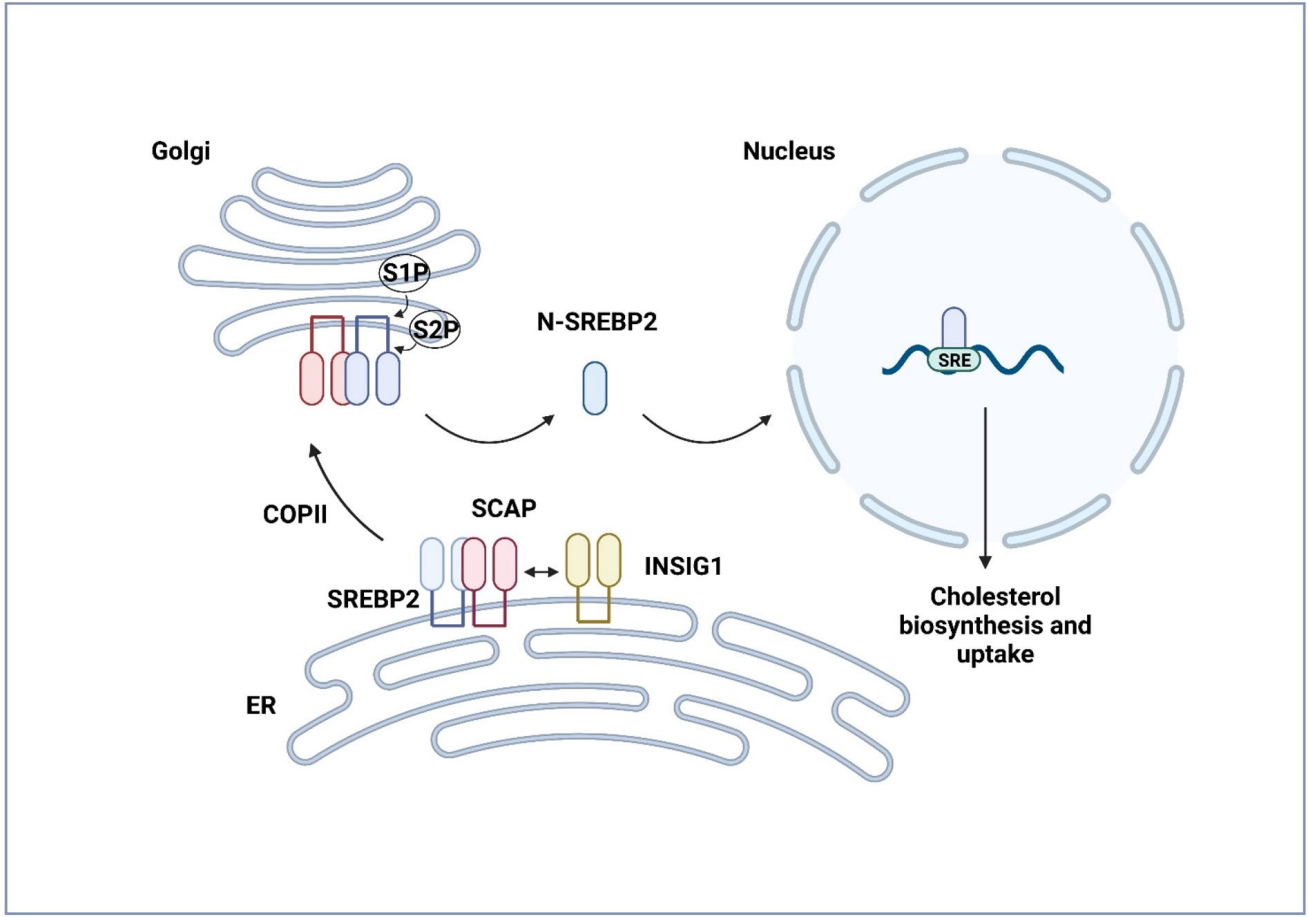
Graphical illustration of sterol regulatory element binding protein 2 (SREBP2) activation pathway. After the complex formed by SREBP2 and SREBP cleavage activating protein (SCAP) detaches from insulin-induced gene 1(INSIG1) the complex locates from endoplasmic reticulum (ER) to Golgi in the Coat Protein Complex II (COP2) coated vesicles. In Golgi, SREBP2 goes through a proteolytic processing by two cleavage enzymes known as membrane-bound transcription factor site-1-protease (S1P) and site-2-protease (S2P). Consequently, the active transcription factor (N-SREBP2) is released, and translocates to the nucleus where it binds to sterol regulating element (SRE) sequences and induces transcription of genes participating in cholesterol biosynthesis and uptake

Activation of SREBP2 is controlled by a cholesterol-dependent transport system between ER and Golgi (Fig. 2). SREBPs locate in the ER as heterodimers with another ER membrane protein, SCAP, which has as a sterol-sensing domain regulating the transport from ER to Golgi (Brown and Goldstein 1999). SCAP functions as an escort protein for SREBP and is essential for SREBP stability (Sever et al. 2003). In a case of sterol depletion, the SCAP/SREBP complex locates to the Golgi apparatus in the coat protein complex II (COP2) coated vesicles (Goldstein et al. 2006). In Golgi, SREBP goes through proteolytic processing by two cleavage enzymes: membrane-bound transcription factor site-1-protease (S1P) and site-2-protease (S2P) (Brown and Goldstein 1999). As a result, an active, soluble N-terminal-cleaved transcription factor of the basic helix-loop-helix leucine zipper family is created (Brown and Goldstein 1997; Goldstein et al. 2006). This structural change allows SREBPs to enter the nucleus as homodimers, bind to sterol regulating element (SRE) sequences and induce gene transcription. On the contrary, when sterol level is high, S1P reaction is blocked and the SCAP/SREBP complex remains in ER.

### Empirical evidence

The ability of INSIG1 to control SREBP2 activity is widely established (Brown and Goldstein 1997; Ouyang et al. 2020). Under normal conditions, increased cholesterol level and SREBP2 processing induce *INSIG1* mRNA expression, which abolishes SREBP2 activity. PXR activation may alter INSIG1 stability as described in KER1. Karpale et al. (2021) observed that activation of SREBP2 was independent from cholesterol level, indicating a perturbation in the normal regulation of SREBP2 pathway possibly mediated by altered INSIG1 function. Similar results have been observed in the liver and intestine of *Insig* knockout mice, where nuclear SREBP and mRNA level of SREBP target genes remain elevated despite overaccumulation of sterols, which should downregulate SREBP activity (Engelking et al. 2005; McFarlane et al. 2014). When INSIG1 proteins are absent, SREBPs are resistant to cholesterol-dependent suppression (McFarlane et al. 2014). In vitro studies have revealed that SREBP transport to nucleus is inhibited by binding of SCAP to INSIG when ER cholesterol level reaches above 5% of total ER lipid level (Radhakrishnan et al. 2008). INSIG depletion allows cleaving of SREBPs despite high cholesterol levels by increasing the threshold that should initiate the negative feedback (McFarlane et al. 2014). Similarly, the excess of cholesterol fails to stabilize INSIG1 in SCAP-deficient cells (Gong et al. 2006). Furthermore, saturation of INSIG1 allows remaining unbound SCAP to transport SREBP2 to Golgi and is also, therefore, resistant to sterol-mediated inhibition of proteolytic processing of SREBP (Yang et al. 2002).

### Known feedback loops influencing KER2

Transcription of INSIG1 is dependent on cholesterol level and activation of SREBPs (Goldstein et al. 2006). *INSIG1* mRNA production is enhanced when cells are sterol depleted or if cleaved SREBP occurs in the nucleus and is prevented when cholesterol accumulates or if inactive SREBP is retained in ER. Quite the opposite, the INSIG1 protein is ubiquitinated and degraded rapidly when cells are sterol depleted. In sterol-accumulated cells, INSIG1 structure is stabilized by SCAP. Moreover, the expression of INSIG1 is reliant on SREBP1c, whose transcription is controlled by insulin (Chen et al. 2004; Goldstein et al. 2006). Low insulin level during fasting leads to repression of SREBP1c and suppression of *Insig1* mRNA. When insulin level increases, SREBP1c processing and *Insig1* mRNA increases concordantly.

Two classes of sterols, cholesterol, and oxysterols, regulate SREBP2 activity by inhibiting the proteolytic processing of SREBP2, which reduces the level of active transcription factor in the nucleus (Goldstein et al. 1979). To inhibit the translocation of SREBP2 to be processed in Golgi, cholesterol binds to SCAP and oxysterols bind to INSIGs, which triggers INSIG to bind to SCAP (Sun et al. 2007). These mechanisms result in conformational changes in SCAP preventing clustering of SREBP with COP2 vesicles that are responsible of the translocation of SREBP to Golgi.

High-fat diet seems to dysregulate SREBP2 activation. Upregulated expression of SREBP2 mRNA and protein has been observed in the livers of mice exposed to high-fat diet (Wu et al. 2013). In accordance with Karpale et al. (2021), increased *Insig1* mRNA level was also observed. On the other hand, in the study by Karpale and colleagues (2021), high-fat diet alone did not induce the expression of SREBP2, but the activation was shown to be PXR dependent.

## KER3 between SREBP2 activation and increased level of PCSK9

### Key event relationship description

PCSK9 is a circulating hepatic protein and has a crucial role in regulating cholesterol level (Seidah et al. 2003; Poirier et al. 2008). The transcription of *PCSK9* is controlled by SREBP2. PCSK9 is expressed mainly in liver, especially during development and it is also present in small intestine, kidney, and brain (Seidah et al. 2003).

### Biological plausibility

SREBP2 acts as a main regulator of *PCSK9* transcription (Horton et al. 2007) via functional sterol regulatory element (SRE) in proximal promoter of *PCSK9* (Li et al. 2009). PCSK9 is secreted by the liver as a zymogen precursor and is autocatalytically cleaved in the ER, releasing the N-terminal prodomain of PCSK9, which further forms a noncovalent bond with the catalytic or C-terminal domain (Seidah 2013). N-terminal prodomain prevents PCSK9 function and to activate, catalytic domain of PCSK9 needs to be released from the prodomain**.**

### Empirical evidence

The role of SREBP2 as the main transcriptional regulator of *Pcsk9* expression is well established in transgenic and S*rebp* knockout mice as well as in vitro studies (Horton et al. 2003; Maxwell et al. 2003; Hyun et al. 2008). As mentioned above, SREBP2 level is highly correlated with plasma cholesterol level, which further reflects to *PCSK9* transcription (Lagace 2014). PCSK9 expression is also increased after PXR activation both in mice and humans and in mouse this was shown to be associated with increased nuclear SREBP2 level (Karpale et al. 2021).

## KER4 between SREBP2 activation and increased level of cholesterol synthesis enzymes

### Key event relationship description

The genes induced by SREBP2 encode enzymes that are involved in different phases of the mevalonate pathway of cholesterol synthesis including 3-hydroxy-3-methylglutaryl-coenzyme (HMGCR) (Hua et al. 1993). HMGCR is the first rate-limiting enzyme in the mevalonate pathway emphasizing its role as a main target for regulation (DeBose-Boyd 2008). HMGCR is localized in ER of all mammalian species studied including human, mouse, rat, and hamster.

### Biological plausibility

SREBP2 induces the expression of several genes involved in cholesterol synthesis and uptake by binding to SREs in the regulatory regions (Horton et al. 2002). Only few SREs in the promoters of these genes are characterized in humans and in some extent in other species (Sharpe and Brown 2013). However, SREs might not be conserved among species.

### Empirical evidence

HMGCR is directly regulated by SREBP2 via gene transcription (Howe et al. 2017). This is supported by the finding that decreased amount of active SREBP2 leads to reduction of mRNA levels of *Hmgcr* both in vivo and in vitro (König et al. 2007). SREBP2-regulated inhibition of mevalonate pathway is a target for cancer therapy and several preclinical studies have shown that modulating SREBP2 activity reflects to the activity of the mevalonate pathway, as reviewed by Xue and coworkers (Xue et al. 2020). Moreover, PXR activation in obese mice yielded in increased expression level of nearly all genes participating in cholesterol synthesis and the effect is predicted to be SREBP2 mediated. (Karpale et al. 2021).

### Known feedforward/feedback loops influencing KER4

In addition to rather slow SREBP2 mediated regulation, HMGCR is more rapidly regulated post-translationally by INSIG1 (Sharpe and Brown 2013). INSIG1 accelerates degradation of HMGCR in ER by combining to the sterol sensing domain of HMGCR (Ouyang et al. 2020). In cellular cholesterol depletion, INSIG1 protein is unstable and unable to bind HMGCR, but when the cholesterol level increases, the structure stabilizes and binds to HMGCR causing the degradation and preventing the further increase of cholesterol level. INSIG1 dysfunction, possibly caused by PXR, induces perturbations in the INSIG1-mediated regulation of HMGCR (Karpale et al. 2021). The effect was observed in obese mice, where HMGCR mRNA and protein levels were increased, despite cholesterol accumulation.

## KER5 between PXR activation and increased activity of SQLE

### Key event relationship description

SQLE, encoded by *SQLE* gene, is the second rate-limiting enzyme in the mevalonate pathway (Hidaka et al. 1990; Nagai et al. 1997; Gill et al. 2011). SQLE is a vital enzyme among eukaryotes (Chua et al. 2020), but it serves as a controller of cholesterol synthesis only in mammals. SQLE is expressed ubiquitously in mammals, yet is the most abundant in esophagus, testis, and liver, the latest serving as the main organ for cholesterol synthesis (Uhlén et al. 2015; Chua et al. 2020). Activation of PXR has been shown to directly activate SQLE (Gwag et al. 2019).

### Biological plausibility

The ability of PXR to bind DR-2 site in *Sqle* gene promoter region was recently identified, indicating that *Sqle* is a direct transcriptional target of PXR (Gwag et al. 2019). Additionally, PXR activation has been observed to increase both mRNA and protein level of SQLE (Gwag et al. 2019; Karpale et al. 2021). On the other hand, *Sqle* is a target gene for SREBP2, which activity is also shown to be controlled by PXR, as described above.

### Empirical evidence

Increased *Sqle* mRNA expression after PXR activation has been observed in few studies (Table 1.) (Gwag et al. 2019; Jiang et al. 2021; Karpale et al. 2021). In addition to mRNA level, Gwag et al. (2019) discovered increased protein level of SQLE both in vivo and in vitro. SQLE was also identified as a direct transcriptional target of PXR (Gwag et al. 2019).

### Known feedforward/feedback loops influencing KER5

SQLE activity is controlled by cholesterol via direct feedback mechanism, abundance of cholesterol causing degradation of SQLE enzyme (Chua et al. 2020). The activation of SQLE transcription is controlled by SREBP2 and its cofactors, and transcriptional activity increases in sterol-depleted conditions. The regulation of SQLE activity is thought to be independent from the rest of the cholesterol synthesis pathway, since the SQLE enzyme adjusts the cholesterol synthesis within the pathway, making it suitable for fast and flexible adapting of cholesterol levels (Gill et al. 2011; Chua et al. 2020).

### Uncertainties and inconsistencies

Long-term treatment with a PXR agonist PCN does not seem to induce *Sqle* mRNA level in male C57BL/6 mice (Zhang et al. 2022). However, the liver samples from PCN-treated mice were collected after 7 days of the last dose, which might cause reversal of PXR activation. Also, long-term treatment may cause alterations in the status of the receptor due to adaptation of liver to external stimuli (Zhang et al. 2022). In accordance with Zhang & co-workers, Ann Barretto et al. (2019) did not observe any changes in *Sqle* regulation in male C57BL/6 mice after 4-day administration of PCN regardless of PXR activation. Increase in cholesterol biosynthesis was not observed either, but previous studies had shown that mice on normal chow diet are resistant to changes in lipid profile, which might be the case in this study as well (Ann Barretto et al. 2019; Hassani-Nezhad-Gashti et al. 2019). On the other hand, effect of PXR activation on SQLE activation and lipid level has been observed in mice on a regular diet in other studies (Gwag et al. 2019; Jiang et al. 2021).

## KER6 between increased level of PCSK9 and increased level of plasma LDL cholesterol

### Key event relationship description

Liver is the main organ clearing LDL from plasma and excreting cholesterol from the body (Spady et al. 1985). Fluctuations in the regulation of the efflux pathway lead to unbalanced homeostasis of sterols. Sufficient hepatic uptake of LDL cholesterol by LDLRs is crucial for regulation of circulating LDL cholesterol level. Decreased LDL uptake by liver caused by downregulated number of LDLRs in plasma membrane of hepatocytes leads to inadequate hepatic clearance of cholesterol. This results in increased level of circulating LDL cholesterol. PCSK9 controls the amount of LDLRs in hepatocytes (Seidah et al. 2014).

### Biological plausibility

In the bloodstream, activated PCSK9 binds to hepatic LDLRs enhancing the lysosomal degradation of the receptors (Seidah 2013). Two different pathways of PCSK9 mediated LDLR degradation have been identified (Lagace 2014). PCSK9 binds to LDLR intracellularly and escorts it from trans-Golgi to lysosomal degradation. Secondly, secreted PCSK9 may bind to the first epidermal-growth factor-like repeat of LDLR on the cell surface leading to internalization of the PCSK9-LDLR-complex in endocytic vesicles.

### Empirical evidence

The role of PCSK9 as a regulator of LDLRs and in development of hypercholesterolemia is well described (Grefhorst et al. 2008; Lambert et al. 2008; Seidah 2013) and overexpression of PCSK9 accumulates LDL in the circulation (Benjannet et al. 2004; Lalanne et al. 2005; Lambert et al. 2006). Enhanced excretion of PCSK9 from liver causes degradation of LDLRs and is resulted in increased plasma LDL levels (Seidah 2013). Discovery of the mechanism has resulted in the development of pharmacological applications and nowadays PCSK9 inhibitors are a common treatment for hypercholesterolemia (Seidah et al. 2014).

### Known feedback loops influencing ***KER6***

In addition to post-transcriptional regulation of LDLRs by PCSK9, transcription of LDLRs is regulated by SREBP2 (Horton et al. 2002). SREBP2 activation increases the expression of LDLRs thereby increasing cholesterol uptake from plasma. Since SREBP2 regulates Pcsk9 at transcriptional level, it induces simultaneous opposite effects on LDLRs by regulating the amount of LDLRs at both transcriptional and post-transcriptional levels. (Rashid et al. 2005).

## KER7 between increased level of cholesterol synthesis enzymes and increased level of plasma LDL cholesterol

### Key event relationship description

In addition to LDL clearance, regulation of cholesterol synthesis is critical in maintaining cholesterol homeostasis (Spady et al. 1985). In mammals, almost all cells synthesize cholesterol (Brown and Sharpe 2016). However, level of plasma cholesterol is dictated by liver cholesterol synthesis. Cholesterol synthesis is a complex process, and it is highly regulated by negative feedback mechanism to avoid cholesterol depletion or excess. Therefore, in cases where the cholesterol synthesis pathway is dysregulated, the process of cholesterol synthesis may persist even in the presence of cholesterol accumulation. This is in contrast to the feedback mechanism, as the accumulation of cholesterol should typically impede the function of the enzymes responsible for maintaining cholesterol homeostasis (Engelking et al. 2005).

### Biological plausibility

**Fig. 3:**
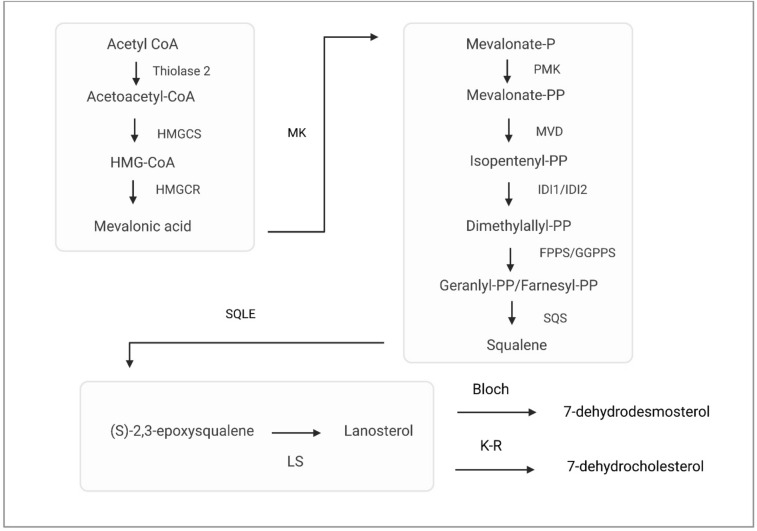
Mevalonate pathway of cholesterol synthesis. First, acetyl-CoA is converted to acetoacetyl-CoA by thiolase 2. Secondly, methylglutaryl-coenzyme A synthase (HMGCS) condenses acetyl-CoA with acetoacetyl-CoA to form HMG-CoA (Brown and Sharpe 2016). Next, HMGCR reduces HMG-CoA to mevalonic acid. Mevalonic acid is phosphorylated to mevalonate-P by mevalonate kinase (MK) and mevalonate-P is further produced to mevalonate-PP by phosphomevalonate kinase (PMK). This is followed by catalyzation of mevalonate-PP to isopentenyl-PP by diphosphomevalonate decarboxylase. Next, dimethylallyl-PP is isomerized by isopentenyl-diphosphate Δ-isomerase (IDI1/IDI2). The isoprenoid-pyrophosphates are condensed to isoprenoid geranyl-PP by farnesyl diphosphate synthase (FPPS) or to farnesyl-PP by geranylgeranyl pyrophosphate synthase (GGPPS) and finally, squalene synthase (SQS) converts farnesyl-PP to squalene. Squalene is converted to (S)-2, 3-epoxysqualene by squalene epoxidase (SQLE) and (S)-2,3-epoxysqualene is further converted to lanosterol and finally to 7-dehydrodesmosterol or 7-dehydrocholesterol via Bloch or Kandutch-Russel (K-R) pathways, respectively

As described above, cholesterol regulates several proteins by enhancing or inhibiting their function depending on the needs of a cell. The first step in the mevalonate pathway of cholesterol synthesis (Fig. 3) is conversion of acetyl coenzyme A to acetoacetyl coenzyme A by thiolase 2 (Brown and Sharpe 2016). Methylglutaryl-coenzyme A synthase is the second enzyme condensing acetyl coenzyme A with acetoacetyl coenzyme A to form 3-hydroxy-3-methylglutaryl coenzyme A. Next, HMGCR reduces the synthetized 3-hydroxy-3-methylglutaryl coenzyme A to mevalonic acid. Mevalonic acid is phosphorylated to mevalonate-P by mevalonate kinase and mevalonate-P is further produced to mevalonate-PP by phosphomevalonate kinase. Next, diphosphomevalonate decarboxylase catalyzes the reaction of mevalonate-PP to isopentenyl-PP, followed by isomerization to dimethylallyl-PP by isopentenyl-diphosphate Δ-isomerase. These isoprenoid-pyrophosphates are then condensed to isoprenoid geranyl-PP by farnesyl diphosphate synthase or to farnesyl-PP by geranylgeranyl pyrophosphate synthase and finally, squalene synthase converts farnesyl-PP to squalene. Then squalene is converted to (S)-2,3-epoxysqualene by SQLE and (S)-2,3-epoxysqualene is further converted to lanosterol and finally to cholesterol via Bloch or Kandutch–Russel pathways. Furthermore, surplus cholesterol undergoes esterification through acyl coenzyme A, resulting in the formation of cholesteryl esters (Luo et al. 2019). These esters serve as a cholesterol reserve within cytosolic lipid droplets or are released as prominent constituents of plasma lipoproteins, such as chylomicrons, very low-density lipoproteins (VLDLs), LDLs, and high-density lipoproteins (HDLs). PXR may induce enzymes participating in hydrolysis of cholesterol esters in the intestine (Helsley et al. 2013).

Due to the criticalness of the enzymes involved in the cholesterol synthesis pathway, their inhibition has yielded several pharmacological applications. For example, the mechanism of action of common LDL cholesterol-lowering drugs, statins, is based on inhibition of HMGCR, which prevents further processing of cholesterol precursors in the mevalonate pathway and therefore decreases plasma cholesterol levels (Sirtori 2014).

### Empirical evidence

In *Insig1* knockout mice, the degradation of the rate-limiting enzyme HMGCR, is prevented, allowing the prolonged continuation of cholesterol synthesis resistant to cholesterol-dependent regulation (Engelking et al. 2005; McFarlane et al. 2014). On the contrary, in *Hmgcr* knockout mice, hepatic cholesterol synthesis is decreased (Nagashima et al. 2012). In addition, increased expression of the second rate-limiting enzyme of cholesterol synthesis, SQLE, results in elevated plasma, total, LDL, and HDL cholesterol in C57BL/6 wild-type, liver-specific Pxr-flox allele-carrying, and PXR-humanized C57BL/6 mice (Gwag et al. 2019).

### Known feedforward/feedback loops influencing KER7

Cholesterol synthesis pathway is highly regulated to avoid fluctuations in cholesterol level. The feedback inhibition pathways controlling SREBP2 and HMGCR activity (KER2 & KER4), lead to downregulation of most of the enzymes in cholesterol synthesis pathway and degradation of HMGCR, respectively (Goldstein et al. 1979; DeBose-Boyd 2008). As a result, cholesterol synthesis decreases.

### Modulating factors

Generally, squalene, an intermediate of cholesterol synthesis, tends to accumulate in the liver in the presence of high cholesterol level due to downregulation of SQLE (Gill et al. 2011). However, decreased squalene level has been observed in mice with PXR-induced hypercholesterolemia (Karpale et al. 2021). The authors suggest that this indicates enhanced squalene metabolism caused by increased SQLE activity mediated by PXR.

Interestingly, Karpale et al. (2021) also showed that PXR activation induces the activation of Kandutsch–Russel pathway in both humans and mice, but the mechanism is unknown. Increased level of plasma and hepatic markers of the pathway and induction of the 24-dehydrocholesterol reductase enzyme was described, leading cholesterol synthesis towards Kandutsch–Russel pathway (Karpale et al. 2021).

## AO: increased plasma LDL cholesterol

### Key event description

The main function of LDL is to provide cholesterol to the cells and, therefore, it has a major role in cholesterol transfer and metabolism (Hevonoja et al. 2000). The LDL particle consists of a core formed of triglycerides and cholesteryl esters and of a surface comprised of phospholipids and a copy of a ApoB-100 lipoprotein. As a consequence of decreased hepatic clearance and overproduction of LDL, the concentration of circulating LDL increases. Next, LDL penetrates the vascular wall, especially on the parts with existing dysfunction or reduced blood flow and accumulates within the intima of arteries (Ziegler et al. 2020). Accumulated LDL particles are oxidized via reactive oxygen species, which triggers an inflammatory response and foam cell formation by macrophages further promoting the inflammatory state in the artery (Huff et al. 2016). Hypercholesterolemia is the most common atherogenic dyslipidemia, and ultimately leads to atherosclerosis characterized by formation of calcified and prone to rupture plaques in arteries, leading to complete obstruction of the artery.

### Modulating factors

Development of hypercholesterolemia involves not only hepatic cholesterol synthesis but also the absorption of biliary and dietary cholesterol in the intestine (Jia et al. 2011). Niemann-Pick C1 Like 1 (NPC1L1) and ATP-binding cassette transporters G5 and G8 (ABCG5/G8) play crucial roles in regulating cholesterol uptake and efflux not only in the intestine but also in the liver and PXR and SREBP2 may contribute to their regulation (Alrefai et al. 2007; Tremblay et al. 2011; Sui et al. 2015; Meng et al. 2019). On the contrary, in obese mice PXR strongly induced hepatic cholesterol synthesis but had no effect on Npc1l1 expression but prompted Abcg5 expression (Karpale et al. 2021). Additionally, PXR activation led to elevated serum hepatic apolipoprotein B levels, reduced intestinal apolipoprotein B48 levels, and did not affect intestinal Apob expression in humans. These findings provide evidence for the repression of intestinal cholesterol absorption and propose that the PXR-induced increase in serum cholesterol occurs independently of intestinal cholesterol absorption and synthesis (Karpale et al. 2021). However, there is other evidence suggesting that also intestinal processes may play a role (Meng et al. 2019; Sui et al. 2021).

## Overall assessment of AOP

Hypercholesterolemia is a major risk factor for vascular damage, cardiovascular diseases, and a critical contributor to global health burden. Several intrinsic and external factors such as environmental pollutants contribute to hypercholesterolemia. To elucidate the unclear mechanism behind chemical-induced hypercholesterolemia we applied an AOP framework for PXR activation leading to hypercholesterolemia. Essentiality of KEs, the biological plausibility and empirical evidence of KERs are described thoroughly in respective KER chapters and summarized in Table 2. Additionally, the quality of evidence rating is summarized in Table 1.

**Table 2:** Overall assessment of evidence supporting the adverse outcome pathway (AOP) based on essentiality of key events (KEs), and biological plausibility and empirical evidence for key event relationships (KERs)

KE	KE description	Support for essentiality of the KE
		Is a downstream KE prevented if an upstream KE is blocked?
MIE: PXR activation	PXR activation was identified as a key triggering event in chemical-induced hypercholesterolemia	PXR activation induces hypercholesterolemia, effects abolished in *Pxr*^*−/−*^ mice (Gwag et al. 2019; Karpale et al. 2021) PXR disturbs the proteolytic machinery controlling INSIG1 stability (KE1) → disrupts the ability of INSIG1 to regulate its targets such as SREBP2 (KE2) and HMGCR (Karpale et al. 2021) Increased SQLE mRNA (Gwag et al. 2019; Jiang et al. 2021; Karpale et al. 2021) and protein (Gwag et al. 2019) levels after PXR activation has been observed (KE5)
KE1: instability of INSIG1	INSIG1 functions as a regulator of cholesterol synthesis and uptake	INSIG1 controls SREBP2 activity (Eberlé et al. 2004). Stabilized INSIG1 prevents SREBP2 activation INSIG1 controls the activity of HMGCR (Sharpe and Brown 2013)
KE2: SREBP2 activation	SREBP2 is the main regulator of cholesterol synthesis pathway	SREBP2 activation induces the transcription of its target genes (KE4) (Brown and Goldstein 1997) SREBP2 is the main controller of PCSK9 (KE3) (Horton et al. 2003)
KE3: increased level of PCSK9	PCSK9 plays a central role in regulating cholesterol levels	The ability of PCSK9 to decrease the amount of LDLRs and, therefore, increase plasma LDL cholesterol (AO) is widely established (Seidah et al. 2014)
KE4: increased level of HMGCR & other cholesterol synthesis enzymes	HMGCR is the 1st rate-limiting enzyme in cholesterol synthesis	Induction of HMGCR activity enhances the further processing of cholesterol precursors in the cholesterol biosynthesis pathway thus increasing plasma cholesterol levels (AO) (Brown and Sharpe 2016)
KE5: SQLE activation	SQLE acts as the 2nd rate-limiting enzyme of cholesterol synthesis	Increased activity of SQLE induces the conversion of squalene to (S)-2,3-epoxysqualene in cholesterol biosynthesis pathway thus increasing plasma cholesterol levels (AO) (Chua et al. 2020)
AO: increased plasma LDL cholesterol	Excess LDL cholesterol accumulates in the vascular walls and is a major contributor in the development of atherosclerotic cardiovascular diseases	

KER	Support for biological plausibility of the KER	Empirical support for the KER
KER1 between PXR activation and dysfunction of INSIG1	PXR causes degradation of INSIG1 protein by an unknown mechanism (Karpale et al. 2021)	Limited results in a single study suggest disturbance of the mechanisms controlling INSIG1 stability (Karpale et al. 2021)
KER2 between INSIG1 instability and SREBP2 activation	Processing of SREBP2 depends on INSIG1 stability, which in turn is controlled by cholesterol level and by the level of active SREBP2 in the nucleus (Goldstein et al. 2006)	It is widely established that degradation of INSIG1 promotes the process of SREBP2 activation (Shimano and Sato 2017)
KER3 between SREBP2 activation and increased level of PCSK9	SREBP2 activates the transcription of PCSK9 via functional SRE in proximal promoter of PCSK9 (Li et al. 2009)	There is great amount of evidence that SREBP2 acts as the main regulator of PCSK9 expression (Horton et al. 2003; Hyun et al. 2008)
KER4 between SREBP2 activation and increased level of cholesterol synthesis enzymes	SREBP2 activates the expression of several genes involved in cholesterol synthesis and uptake by binding to SREs in the promoter regions of the genes which enhances the transcription (Horton et al. 2002)	SREBP2-mediates activation of the entire pathway of cholesterol synthesis (Horton et al. 2003)
KER5 between PXR activation and increased activity of SQLE	PXR binds to DR-2 site in *SQLE* gene promoter region (Gwag et al. 2019) and increases mRNA and protein levels of SQLE (Gwag et al. 2019; Karpale et al. 2021) *SQLE* is a target gene for SREBP2 (Chua et al. 2020), which activity is controlled by PXR (Karpale et al. 2021)	The ability of PXR to bind *Sqle* gene promoter region is observed in single study (Gwag et al. 2019) and there is some experimental evidence that mRNA and protein levels of SQLE are increased after PXR activation (Gwag et al. 2019; Jiang et al. 2021; Karpale et al. 2021) On the contrary, no changes in *Sqle* regulation after PXR activation have been observed (Ann Barretto et al. 2019; Zhang et al. 2022)
KER6 between increased level of PCSK9 and increased level of plasma LDL cholesterol	Activated PCSK9 binds to hepatic LDLRs enhancing the lysosomal degradation of the receptors (Seidah 2013). Decreased LDL uptake by liver caused by decreased amount of LDL receptors in plasma membrane of hepatocytes therefore leads to inadequate hepatic clearance of cholesterol resulting in increased level of circulating LDL cholesterol (Spady et al. 1985)	There is a great amount of evidence that degradation of hepatic LDLRs by PCKS9 binding increases the level of circulating LDL cholesterol (Seidah et al. 2014)
KER7 between increased level of cholesterol synthesis enzymes and increased level of plasma LDL cholesterol	Increased cholesterol synthesis due to induction of enzymes participating in the biosynthesis of cholesterol, especially rate-limiting enzymes HMGCR and SQLE, results in increased plasma cholesterol levels (Gwag et al. 2019; Karpale et al. 2021)	There is solid evidence that increased activity of cholesterol synthesis enzymes increases circulating cholesterol levels, including LDL cholesterol (Brown and Sharpe 2016)

*PXR* pregnane X receptor, *INSIG1* insulin-induced gene 1, *SREBP2* sterol regulatory element binding protein 2, *SRE* sterol regulatory element, *HMGCR* 3-hydroxy-3-methylglutaryl coenzyme a reductase, *SQLE* Squalene epoxidase, *PCSK9* proprotein convertase subtilisin kexin type 9, *LDLR* Low density lipoprotein receptor

The first KER focuses on the effect of PXR activation on INSIG1 function. As INSIG1 controls cholesterol synthesis and uptake, an aberration in expression or function of INSIG1 is causal to lipid disorders (Ouyang et al. 2020). Even though PXR has been shown to bind the DR-4 site in the upstream promoter region of *Insig1* (Roth et al. 2008) and the recent results suggest that PXR may regulate the translation or protein stability of INSIG1 (Karpale et al. 2021), further research is needed to determine the underlying mechanism.

Disturbed function of INSIG1 is a critical key point in the cholesterol synthesis pathway, since INSIG1 controls the activation of SREBP2. Activation of SREBP-pathway activates enzymes participating in cholesterol synthesis as well as the PCKS9 enzyme. In addition to PXR-mediated activation of SREBP-pathway, PXR affects directly on cholesterol synthesis pathway by regulating *SQLE* transcription. Together these mechanisms lead to increased cholesterol synthesis and decreased uptake of LDL by liver and finally to increased plasma LDL cholesterol (AO).

This AOP is a plausible description of the link between PXR activation and hypercholesterolemia and combines information from reviews and published data from in vivo and in vitro studies. Evidence regarding KERs 1 and 5 is scarce, and more studies are needed to better understand the relationships. On the other hand, the rest of the KERs are well established and supported by solid background of biological knowledge and empirical evidence. It must be noted that AOPs aim to simplify the toxicity pathways and focus merely on the essential events, as done in the current AOP. We acknowledge that all the KEs and KERs are also affected by other mechanism not described in the AOP.

Even though most of the described molecular interactions occur in various tissues and the KEs are applicable in both sexes through all life stages across taxa, we choose to limit the AOP to consider liver due to its criticalness in cholesterol metabolism and in chemical-induced hypercholesterolemia. This also reflects the predominant expression of PXR in the liver.

As a conclusion, the AOP illustrates how PXR potentially disturbs the regulation of cholesterol synthesis and LDL metabolism. The AOP was developed to offer an insight into the molecular mechanism of PXR mediated hypercholesterolemia, since the mechanisms of drug- and chemical-induced hypercholesterolemia are poorly understood. In the future, the AOP can be elaborated and possibly be included in the AOP-Wiki, which could promote safety assessment.

## Data Availability

Data sharing not applicable to this manuscript as no datasets were created or analyzed during the current study.
